# Usability in the admission monitoring system of an emergency room

**DOI:** 10.11606/s1518-8787.2021055003475

**Published:** 2021-12-09

**Authors:** Hertaline Menezes do Nascimento Rocha, Ester Batista do Nascimento, Laryssa Carvalho dos Santos, Guilherme Viturino Alves, Anny Giselly Milhome da Costa Farre, Valter Joviniano de Santana-Filho

**Affiliations:** I Universidade Federal de Sergipe Programa de Pós-Graduação em Ciências da Saúde Aracaju SE Brasil Universidade Federal de Sergipe. Programa de Pós-Graduação em Ciências da Saúde. Aracaju, SE, Brasil; II Universidade Federal de Sergipe Departamento de Enfermagem Lagarto SE Brasil Universidade Federal de Sergipe. Departamento de Enfermagem. Lagarto, SE, Brasil; III Universidade Federal de Sergipe Programa de Pós-Graduação em Ciência da Computação São Cristóvão SE Brasil Universidade Federal de Sergipe. Programa de Pós-Graduação em Ciência da Computação. São Cristóvão, SE, Brasil

**Keywords:** Database Management Systems, User-Centered Design, Information Literacy, Emergency Medical Services, Admitting Department, Hospital

## Abstract

**OBJECTIVE:**

To develop and evaluate the usability of the admission monitoring system in an emergency room.

**METHODS:**

This applied research intends to develop a software product and evaluate its usability. The development followed four stages: systematic review, structuring of the system framework, construction of system forms, and evaluation of the information generated. In the evaluation, the experts simulated the use of the system by inserting data from a fictitious medical record. We measured usability using the System Usability Scale (SUS). Scores and scores were calculated individually and globally. We propose these evaluation standards: worst case scenario, poor, average, good, excellent, and best-case scenario.

**RESULTS:**

The *Sistema de Informação e Monitoramento das Internações em Pronto-Socorro* (SIMIPS - Information and Monitoring System for Emergency Room Admissions) monitors the epidemiological profile of admissions to the emergency room, time management, clinical deterioration, incidence of adverse events, and human resource management. The usability of SIMIPS, evaluated by 17 experts, reached the SUS Score 86.5 (best case scenario), and some suggestions for modifications were accepted.

**CONCLUSIONS:**

We consider SIMIPS an easy-to-use tool, with real importance in the management of emergencies in view of overcrowding and congestion problems faced in Brazil.

## INTRODUCTION

The overcrowding in emergency room (ER) has become a global problem that affects health systems and patient safety^1,2^. The main cause of this problem is known as hospitalization in the ER or *boarding*^2^, being the patient remaining in the sector due to lack of hospital beds, after the decision to admit the patient^5^.

Patients *“on boarding”* do not receive the necessary care they would receive in the wards. Therefore, they remain vulnerable^4,10^, and may suffer adverse events, such as delayed administration of medication^6,11,12^ and increased mortality^13,14^

Patients who spend more time in emergency rooms are those who need beds in the medical clinic and/or those who need more advanced care technologies given the high degree of dependence, directly reflecting in the increase of hospital stay and, consequently, in costs^9^.

The Federal Court of Auditors reported in the “*Assistência hospitalar no Sistema Único de Saúde* (SUS)” (Hospital care in the Unified Health System (SUS)), that Brazil has overcrowded public hospital services. In hospitals, patients are admitted in the emergency room corridors, on stretchers, chairs, or benches. According to the document, 64% of emergency hospitals were permanently overcrowded, 19% were often overcrowded, 10% were rarely overcrowded and only 6% of hospitals were never overcrowded^15^.

The same phenomenon occurs in *Unidades de Pronto Atendimento* (UPA - Emergency Care Units), where the frequent hospitalizations of patients represent a distortion of the purpose and possibilities of care for these services^16^.

When hospitalized in these overcrowded units, patients wait for beds, whereby monitor time, prioritization of cases, distribution of care, and assistance risks come across. So we must have a monitoring hospitalization system, for quantitatively and qualitatively evaluation. This study intends to monitor each hospitalization completely, in a clear, fast, and accessible way.

In this scenario, information technology (IT) is a great ally in the health area, seeking new strategies and practical solutions to care and management problems through multiple analyzes of collected data^17^ and using various tools that support the structuring and organization of data and information with reliability, completeness, and accuracy. This facilitates real-time and/or remote storage, processing, access, and sharing, either by the various professionals involved in care, or by the patient/user^18^.

Studies show that the proper use of technological resources in health can contribute to increase the prevention of chronic diseases, reduce risk factors and improve quality and life expectancy, in addition to reducing the need for medical care and associated costs, benefiting the entire health system^19^.

The use of *Sistemas de Informação em Saúde* (SIS - Health Information Systems) in public health management, such as the *Sistema de Informação Hospitalar* (SIH/SUS - Hospital Information System), confirms its relevance for epidemiological surveillance, whether in the diagnosis of the situation, or in the assessment of actions and impact of public policies on the health status of the population^20^.

The current report “*Síntese de evidências para políticas de saúde*” (“Synthesis of evidence for health policies”), published by the Ministry of Health of Brazil^21^, reinforces the use of technologies as a systemic management option to avoid congestion and overcrowding in emergency rooms.

Therefore, we intend to develop and evaluate the usability of a monitoring system for hospital admissions in the emergency room. We assume that this tool can contribute to the processes of improving the quality of care and safety of hospitalized patients and generate management reports that support the development of effective strategies in order to mitigate and deal with problems.

## METHODS

This is an applied research, with the development of the *Sistema de Informação e Monitoramento das Internações em Pronto-Socorro* (SIMIPS), on behalf of the *Instituto Nacional da Propriedade Industrial National Institute of Industrial Property* (INPI - National Institute of Industrial Property) BR512019002197-5. Specialist nurses evaluated the usability, with a predominantly quantitative approach to data. The project was approved by the ethics committee CAAE 17039019.1.0000.5546, opinion 4.168.891.

### System Development

The software construction process followed four steps: 1st) Elaboration of a systematic review with applied research registered in electronic databases (PUBMED, SCOPUS, CINAHAL, *Web of Science*) to gather evidence on hospitalization in the emergency room (*boarding*); 2nd) Structuring the system framework; 3rd) Construction of system forms; 4th) Evaluation of the information generated by the system with crossing information reports

The software development followed the model proposed by Sommerville^22^, using a systematic approach based on the principle of four fundamental activities: Specification; Development; Software Validation, and Evolution.

We used the MVC architecture (Model, View, and Controller) pattern, which allows the project to be worked in layers, facilitating maintenance since the layers have well-defined roles. The Personal Home Page (PHP) programming language generated dynamic content and handled Structured Query Language the database (SQL). Application fields AD 02 and SD 01 and programs GI01 – Information Manager were used; GI04 – Report generator; IA01 - Artificial intelligence; IA02 – Expert Systems.

### Usability Assessment

The *System Usability Scale* (SUS) carried out the usability evaluation of the system, mainly proposed for the evaluation of two web application aspects, learning capacity and usability^23^. We generated Portuguese questionnaires using the *Free, Multilingual System Usability Scale Questionnaire Generator*, resulting in the following questions.

1. I would use SIMIPS often;
2. I think SIMIPS is unnecessarily complex;
3. I think SIMIPS is easy to use;
4. I think I would need technical support to be able to use SIMIPS;
5. I think that the various functions of SIMIPS are very well integrated;
6. I think SIMIPS is inconstant;
7. I imagine most people would learn to use SIMIPS a lot quickly
8. I think SIMIPS is quite uncomfortable to use;
9. I felt very confident using SIMIPS;
10. I need to learn a lot of things before I could use SIMIPS.

The SUS questionnaire mixed positive (odd questions) and negative (even questions) items. In each question, the evaluator expressed the magnitude of their agreement, using a 5-point Likert scale with statements going to disagree strongly (1) to fully agree (5).

Researchers added one more objective question, which was excluded from the SUS score because it was not part of the original: Question 11. Is SIMIPS a tool that facilitates ER management? And one more free open question: Question 12. Do you have suggestions to improve SIMIPS?

The selection of evaluators took into account the scores in at least two criteria, focusing on the care and management areas of the emergency room and/or patient safety and/or hospital quality. We observed the following criteria: being a nurse, master, or PhD with a thesis or dissertation in the areas; being the author/co-author of a scientific article in a peer-reviewed journal; participate/or have participated in research groups/projects; have at least one year of experience in care or management practice; have at least one year teaching experience in care or management practice.

Nurses were considered key professionals to use this system, as they are constantly feeding the information systems in emergency rooms and managing patient flows.

The selection of evaluators was carried out browsing in the *Plataforma Lattes* (Lattes Platform), on behalf of the *Conselho Nacional de Desenvolvimento Científico e Tecnológico* (CNPq - National Council for Scientific, and Technological Development) (*Currículo Lattes e Diretório de Grupos de Pesquisa*/ Lattes Curriculum and Directory of Research Groups) and snowball sampling. The evaluators were contacted via e-mail, receiving an invitation letter in June 2020. After formal acceptance with digital signature of the Informed Consent Term (TCLE), they were registered in SIMIPS in the User role Administrator. Invitations were sent weekly up to three times, following a reminder and the deadline for the complete return of the evaluation, ending on August 31, 2020.

All participants received a written tutorial on using the system and the medical record of a fictitious patient to register in the system and monitor. The objective was to simulate the system power supply as close to reality, soon after the evaluators answered the SIMIPS questionnaire on usability.

The evaluators were named with the letter J, followed by a numerical sequence, and the SUS questionnaire questions, named with the letter Q followed by the numerical sequence.

Quantitative data were tabulated and analyzed using the Microsoft Excel® program. Numerical variables were expressed as a measure of central tendency (mean and median) and a measure of dispersion (standard deviation). We considered categorical variables, in absolute and relative frequencies.

The individual SUS score was calculated according to Brooke^23^ in which, for odd items, the individual score is the grade received minus 1 and, for even items, the score is obtained after subtracting 5 from the grade received. Finally, multiply the sum of all scores by 2.5. The total SUS score was calculated by obtaining the mean score for each item and multiplying the sum of all scores by 2.5.

After calculating the total SUS score, the system was classified as follows: from 13 to 20.5 (worst case scenario); from 21 to 38.5 (poor); 39 to 52.5 (average); from 53 to 73.5 (good); from 74 to 85.5 (excellent); and from 86 to 100 (best case scenario)^24^.

## RESULTS

The literature review showed us that hospitalization in the ER could cause a reduction in the quality of care and omission of care^6,12^, increased mortality and increased length of hospital stay^25,26^, vulnerability to the occurrence of adverse events^5,7,11,12,14^, reduced quality and safety of nursing care provided to the patient^13,27^ and could negatively contribute to the psychosocial experiences of nurses^27^.

Therefore, we incorporated literature information related and complementary to the findings regarding to:

- Entry information: patient registration information;
- Initial assessment: Risk classification data, initial medical assessment, past health history anamnesis, Charlson Index (ICC), and Degree of Dependence on Nursing Care (GDCE) classification by the Patient Classification System (SCP), proposed by Perroca and Gaidzinski^28^;
- Monitoring of patient movements: Data on intra-hospital transfers (inpatient areas or care escalation within the emergency room) and departures (discharge, death, extra-hospital transfer, and evasion);
- Management data: Data on the occupancy rate of the emergency room and inpatient sectors, in addition to the length of stay in the ER (from reception to risk classification, duration of risk classification, from this classification to the first service, then until the decision for admission, the length of stay within the ER, the length of stay in the clinics and the total length of stay);
- Ongoing evaluation: Data on GDCE and *National Early Warning Score* (NEWS)^29^;
- Adverse Event Monitoring: Adverse Event Identification (AE) is done by trackers, identified by the *Institute for Healthcare Improvement*^30^ they are categorized by the *National Coordinating Council for Medication Error Reporting and Prevention* (NCC MERP) *scale*^31^;
- Management reports: information, presented through tables, in which it is possible to obtain the individual patient report, the general report by time period, general report of hospitalization times, and total hospitalization time, obtained through graphs with the crossing of the variables AE *versus* Patient, AE *versus* ICC, AE *versus* SCP, AE *versus* NEWS, AE *versus* ER hospitalizations, AE *versus* Total hospitalizations, AE *versus* Deaths, AE *versus* Readmissions, Death *versus* ER hospitalizations, Deaths *versus* readmissions, Rate of occupation of the ER *versus* AE.

**Figure 1:**
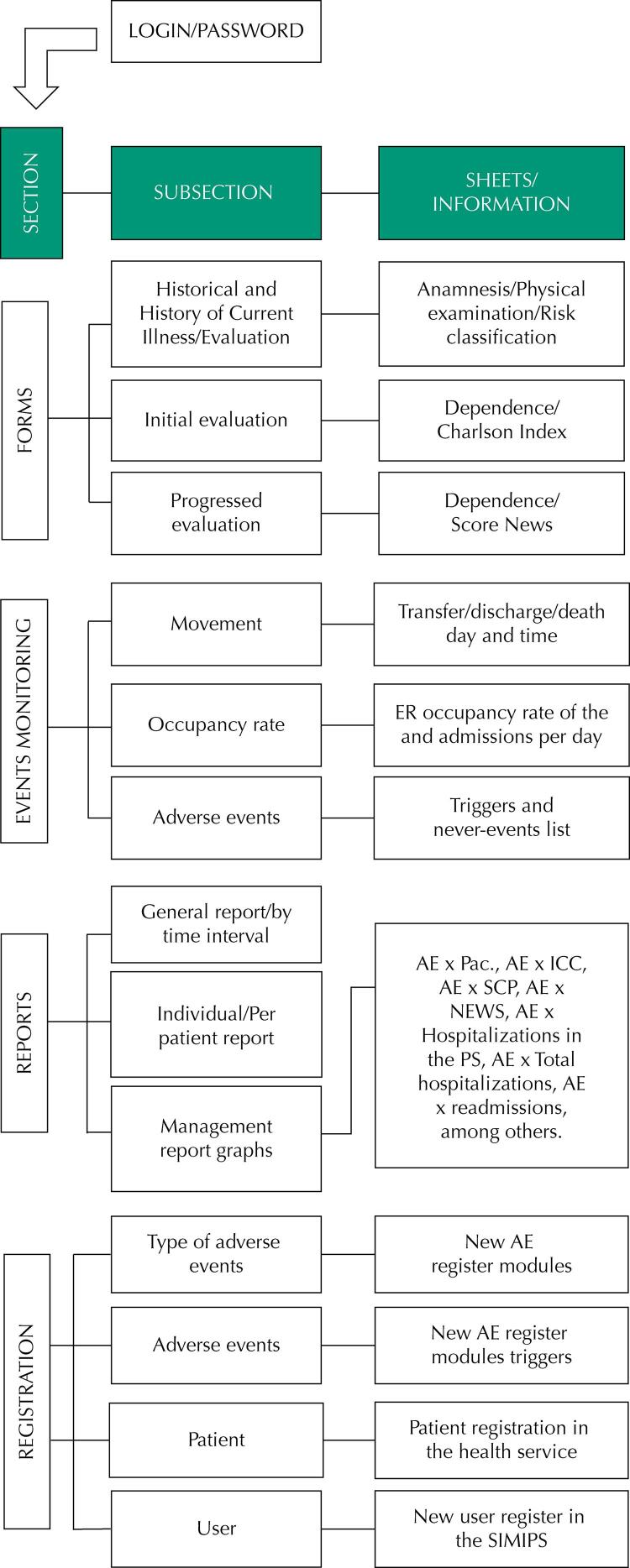
SIMIPS framework.

This version of the system was designed so that the data are fed in a secondary way by a professional from the assistance and management of the emergency room, and the framework of the system was built in subdivisions of sections, subsections, and information sheets. Figure 1 shows the SIMIPS framework.

**Figure 2:**
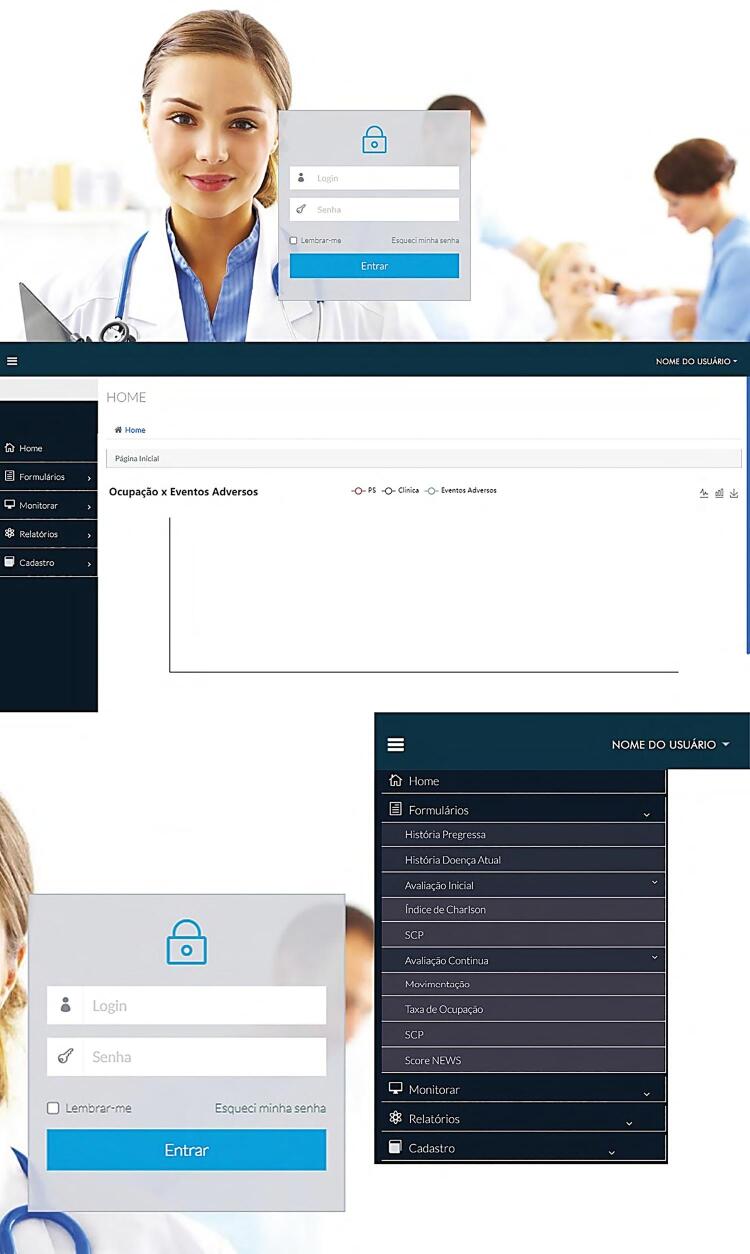
Layout in desktop and mobile formats.

SIMIPS has a responsive technology that adapts the layout to the size of the device used (mobile phone, tablet or *desktop*) to the system be easily loaded. Figure 2 shows the home screen layout in desktop and mobile formats.

Inter. PS: hospitalization in the emergency room; SCP: Patient Classification System.

**Figure 3:**
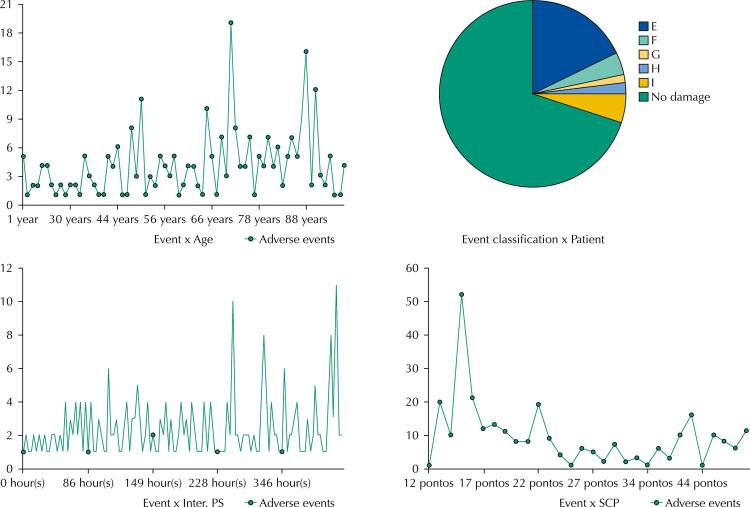
Examples of reports generated by SIMIPS (Information and Monitoring System for Emergency Room Admissions).

With the information inserted in the system, it is possible to carry out comprehensive monitoring of the epidemiological profile of emergency room admissions, the management of time within the unit during hospitalization, the escalation of the hospitalized patient as a result of clinical deterioration, the incidence and severity of events adverse effects, besides favoring a nursing dimension that is adequate to the care needs of inpatients. All this information can be seen in the form of graphs with crossings of covariates in the reports section. Figure 3 shows some of the reports that can be generated by SIMIPS.

We invited 43 evaluators who met the eligibility criteria and agreed to participate in the survey 17 in order to measure the usability of the system. They all completed the evaluation within the established deadline.

In the individual assessment, 59% (n = 10) rated SIMIPS as best case scenario, 35% (n = 6) rated it as excellent and 6% (n = 1) as good. Then we calculated the final score with the average of all results and obtained a score of 86.5, classifying the software as the best case scenario.

The characterization of the evaluators and the classification by the usability questionnaire are described in Table 1. The values assigned to each question in the questionnaire are shown in Table 2. We added question 11 (Q11) to the table given the researchers demands.

**Table 1:** Characterization of the evaluators that managed the Information System and Monitoring of Hospitalizations in Emergency Room and Classification by the Usability Questionnaire (n = 17).

Age (years)	
Mean (DP)	34 (7)
Median (Min-Max)	36 (26–53)
Sex	n (%)
Female	13 (76%)
Male	4 (24%)
Title	n (%)
Master	8 (47%)
PhD	4 (24%)
Specialist	5 (30%)
Professional profile	n (%)
Teacher	4 (29%)
Assistant	11 (65%)
Teacher/Assistant	2 (11%)
Score (criteria)	n (%)
2	6 (35%)
3	3 (18%)
4	4 (24%)
5	2 (11%)
6	1 (6%)
7	1 (6%)
Classification	n (%)
86 to 100 (best case scenario)	10 (59%)
74 to 85.5 (excellent)	6 (35%)
53 to 73.5 (good)	1 (6%)
39 to 52.5 (average)	0
21 to 38.5 (poor)	0
20.5 (worst case scenario)	0
General classification	Score sus total
	86,5 (best case scenario)

**Table 2:** Individual distribution of responses to the Usability Questionnaire.

JUDGE	Q1	Q2	Q3	Q4	Q5	Q6	Q7	Q8	Q9	Q10	Q11
J1	5	1	4	2	4	1	5	1	4	1	5
J2	5	1	5	2	5	2	5	1	5	1	5
J3	5	1	5	1	5	2	4	1	5	1	5
J4	5	2	5	3	4	2	5	2	4	1	5
J5	5	1	4	2	4	1	5	1	4	1	5
J6	5	1	5	4	4	1	5	1	5	1	5
J7	5	2	4	1	5	1	5	1	5	1	5
J8	4	1	4	2	4	2	4	5	5	1	4
J9	5	1	5	2	5	1	5	1	5	2	5
J10	5	2	4	1	4	1	5	2	4	2	5
J11	5	1	5	1	4	1	3	2	4	1	5
J12	4	1	2	1	4	2	4	2	4	1	5
J13	5	2	4	4	5	1	5	2	4	3	5
J14	4	3	3	2	5	2	2	1	4	3	4
J15	4	2	4	3	5	1	3	3	5	1	5
J16	5	1	5	2	5	1	5	1	5	1	5
J17	5	1	5	2	5	2	5	1	5	1	5

In Q11, we asked whether SIMIPS is a tool that facilitates the management of the ER, the answers were I totally agree (n = 15) and partially agree (n = 2).

In response to the last question, we got suggestions for improvements to SIMIPS related to: 1) making the layout more attractive; 2) integrate the hospital information system to facilitate the filling in of information such as vital signs, socioeconomic data, movements, and length of stay whenever possible; 3) to insert an autocomplete filter for CID 10; 4) Insert the option to preview the completed form, before saving, and insert the option “edit” and 5) Put on the home page the list with the names of patients hospitalized at the time, instead of the occupancy rate x events graph adverse effects.

## DISCUSSION

The *Sistemas de Informação em Saúde* (SIS) are intended to help improve the quality of care provided to patients and health professionals, as well as health management through the analysis of costs, benefits, and the reduction of medical errors^32^.

So SIMIPS was conceived as a local (institutional) information system, but with the possibility of being integrated with other local information systems, generating regional databases. Moreover, it generates reports that can be inserted into local and regional information banks and national.

An implicit assumption in the development of a Hospital Information System is the ability to provide complete, accurate, and timely data, so that the professional can perform their task with higher quality at a better cost/benefit ratio^17^.

SIMIPS had its usability evaluated by nurses with a predominance of masters (n = 8), working in emergency care (n = 11), and who met more than 2 inclusion criteria (n = 11), demonstrating scientific capacity and technique of the recruited evaluators.

The SUS questionnaire assessed the usability of SIMIPS through user perception, being classified as best case scenario (score 85.6) in the total assessment and not obtaining any negative assessment (average, poor, and worst case scenario).

When analyzing the users’ responses in the two factors listed by SUS, usability (questions 1, 2, 3, 5, 6, 7, 8, and 9) and learning (questions 4 and 10), we observed in the negative questions 2, 6 and 8, a predominance of strongly disagree and partially disagree responses (Q2 n = 16, Q6 n = 17 and Q8 n = 15). Only one user strongly agreed that SIMIPS would be very uncomfortable to use. In the positive usability questions (1, 3, 5, 7 and 9) there was a predominance of the answers I fully agree and partially agree (Q1 n = 17, Q3 n = 15, Q5 n = 17, Q7 n = 14, Q9 n = 17).

The answers strongly agree and partially agree also predominated (Q4 n = 13 and Q10 n = 15), denoting that SIMIPS can be implemented in an emergency room with higher possibility of acceptance in questions that evaluated the ease of learning about SIMIPS.

The implementation of an Electronic Health System is challenging, as it causes changes and can arouse resistance. Therefore, the information flow must be developed to facilitate its use, understanding, and communication by the team^33^.

The predominance of concordant answers for the positive questions and discordant for the negative ones, both in terms of usability and learning, suggest as easy acceptance of SIMIPS as a management tool, since it was also pointed out by all evaluators that the software facilitates the management of the ER.

Considering that SIMIPS is a new tool, it has some limitations, such as secondary filling, which requires the adhesion of the health team and emergency room managers, a specific sector that is characterized by the intense flow of information and patients. However, in institutions already capable to manage information systems, such as the electronic medical record, it is possible to integrate that information into SIMIPS, speeding up its filling. Another limitation of the system relates to pediatric emergencies usage, given it was developed for use in adult emergency rooms.

However, there was no mention in the literature of another SIS or monitoring system that aims to evaluate and manage hospitalizations in the ER, highlighting the innovative character of SIMIPS. Therefore, the use of SIMIPS as a monitoring tool can support future research on the impact of prolonged stay in the emergency room, on the management of human, material resources, beds, and on the quality of life and work of nursing professionals.

As a management tool, the monitoring generated by SIMIPS will present quality indicators of care in the emergency room for hospitalized patients. Besides, it provides users’ assessment of hospitalization services, guiding changes in work processes aimed at patient safety.

## CONCLUSION

SIMIPS was considered by the evaluators to be an easy-to-use and important tool in the management of the emergency room. However, some adjustments were pointed out to improve the ability to receive information that improves the agility in filling out and managing information. It is important to emphasize that SIMIPS was developed for use in adult ER and that adaptations will be necessary for usage in pediatrics and regional specificities.

However, the initial project generated management data and care indicators that can guide actions to face overcrowding and hospitalizations in emergency rooms, proposing strategies to mitigate the effects of overcrowding on the quality of care.
