# Supplementary material for: Usability in the admission monitoring system of an emergency room
Source: Rev Saude Publica. 2021 Dec 9;55:113. doi: 10.11606/s1518-8787.2021055003475 (PMC8664066; doi:10.11606/s1518-8787.2021055003475)
Supplement: Translation

# Translation: Usabilidade de um sistema de monitoramento das internações em pronto-socorro

**Authors:** Hertaline Menezes do Nascimento Rocha, Ester Batista do Nascimento, Laryssa Carvalho dos Santos, Guilherme Viturino Alves, Anny Giselly Milhome da Costa Farre, Valter Joviniano de Santana-Filho

**Affiliations:** I Brasil Universidade Federal de Sergipe. Programa de Pós-Graduação em Ciências da Saúde. Aracaju, SE, Brasil; II Brasil Universidade Federal de Sergipe. Departamento de Enfermagem. Lagarto, SE, Brasil; III Brasil Universidade Federal de Sergipe. Programa de Pós-Graduação em Ciência da Computação. São Cristóvão, SE, Brasil

**Keywords:** Sistemas de Gerenciamento de Base de Dados, Design Centrado no Usuário, Competência em Informação, Serviços Médicos de Emergência, Serviço Hospitalar de Admissão de Pacientes

## Abstract

**OBJETIVO:**

Desenvolver e avaliar a usabilidade de um sistema de monitoramento das internações em pronto-socorro.

**MÉTODOS:**

Trata-se de uma pesquisa aplicada, com desenvolvimento de um produto de software e avaliação de sua usabilidade por enfermeiros especialistas. O desenvolvimento seguiu quatro etapas: revisão sistemática, estruturação do arcabouço do sistema, construção dos formulários do sistema e avaliação das informações geradas. Na avaliação, os especialistas simularam a utilização do sistema com a inserção de dados de um prontuário fictício, e a usabilidade foi medida pelo *System Usability Scale* (SUS). A pontuação e os escores foram calculados de forma individual e global. O sistema foi avaliado nos padrões: pior imaginável, pobre, mediano, bom, excelente e melhor imaginável.

**RESULTADOS:**

O Sistema de Informação e Monitoramento das Internações em Pronto-Socorro (SIMIPS) realiza o monitoramento do perfil epidemiológico das internações no pronto-socorro, gestão de tempo, deterioração clínica, incidência de eventos adversos e gestão de recursos humanos. A usabilidade do SIMIPS, avaliada por 17 especialistas, atingiu o Score SUS 86,5 (melhor imaginável), e algumas sugestões de modificações foram acatadas.

**CONCLUSÕES:**

O SIMIPS foi avaliado como uma ferramenta de fácil utilização, com real importância na gestão das urgências frente aos problemas de superlotação e congestão, enfrentados no Brasil.

## INTRODUÇÃO

A superlotação dos Prontos-socorros (PS) nas últimas décadas tornou-se um problema mundial que afeta os sistemas de saúde e a segurança dos pacientes^1,2^. A principal causa desse problema é conhecida como internação no PS ou *boarding*^2^, definida como a permanência dos pacientes no setor por falta de leitos hospitalares, após a decisão de internar o paciente^5^.

Os pacientes *“on boarding”* não recebem os cuidados necessários que receberiam nas enfermarias e, portanto, estão mais vulneráveis^4,10^, podendo sofrer eventos adversos, como atraso na administração de medicamentos^6,11,12^ e aumento da mortalidade^13,14^.

Os pacientes que passam mais tempo internados nos prontos-socorros são aqueles que necessitam de leitos de clínica médica e/ou aqueles que, devido ao alto grau de dependência, precisam de tecnologias de cuidado mais avançadas, o que reflete diretamente no aumento do tempo de permanência hospitalar e, consequentemente, nos custos hospitalares^9^.

O relatório “Assistência hospitalar no Sistema Único de Saúde (SUS)”, do Tribunal de Contas da União, apresentou o Brasil como um país com serviços públicos hospitalares superlotados, nos quais os pacientes são internados nos corredores do pronto-socorro, em macas, cadeiras ou bancos. De acordo com o documento, 64% dos hospitais de urgência encontravam-se superlotados permanentemente, 19% ficavam muitas vezes superlotados, 10% lotavam poucas vezes e apenas 6% dos hospitais nunca superlotavam^15^.

O mesmo fenômeno ocorre em Unidades de Pronto Atendimento (UPA), onde as frequentes internações de pacientes representam uma distorção da finalidade e das possibilidades de cuidado desses serviços^16^.

Ao serem internados nessas unidades superlotadas, os pacientes adentram um fluxo de espera por leitos, no qual os profissionais da saúde e gestores têm dificuldades para monitorar o tempo, priorização de casos, distribuição de cuidados e riscos assistenciais. Torna-se, então, necessário um sistema de monitoramento das internações, quantitativa e qualitativamente, que vise estudar e acompanhar cada internação até o seu desfecho, de forma clara, rápida e acessível.

Nesse cenário, a tecnologia da informação (TI) é uma grande aliada na área da saúde, buscando novas estratégias e soluções práticas de problemas assistenciais e gerenciais por meio de múltiplas análises de dados coletados^17^ e utilizando diversas ferramentas que suportam a estruturação e a organização dos dados e informações com confiabilidade, completude e exatidão. Isto possibilita o armazenamento, processamento, acesso e compartilhamento em tempo real e/ou remoto, seja pelos diversos profissionais envolvidos na assistência, seja pelo próprio paciente/usuário^18^.

Estudos apontam que a utilização adequada dos recursos tecnológicos na saúde pode contribuir para aumentar a prevenção de doenças crônicas, reduzir fatores de risco e melhorar a qualidade e a expectativa de vida, além de reduzir a necessidade de cuidados médicos e custos associados, beneficiando todo o sistema de saúde^19^.

A utilização de Sistemas de Informação em Saúde (SIS) na gestão da saúde pública, como o Sistema de Informação Hospitalar (SIH/SUS), confirma sua relevância para a vigilância epidemiológica, seja no diagnóstico da situação, seja na avaliação das ações e do impacto das políticas públicas no estado de saúde da população^20^.

O atual relatório “Síntese de evidências para políticas de saúde”, publicado pelo Ministério da Saúde do Brasil^21^, reforça a utilização de tecnologias como opção de gestão sistêmica para evitar a congestão e superlotação dos prontos-socorros.

Sendo assim, o objetivo desse estudo foi desenvolver e avaliar a usabilidade de um sistema de monitoramento das internações em pronto-socorro. Pressupõe-se que essa ferramenta possa contribuir para os processos de melhoria da qualidade do cuidado e segurança dos pacientes internados, bem como gerar relatórios de gestão que subsidiem a elaboração de estratégias eficazes de mitigação e enfrentamento desse problema.

## MÉTODOS

Trata-se de uma pesquisa aplicada, com o desenvolvimento do Sistema de Informação e Monitoramento das Internações em Pronto-Socorro (SIMIPS), registrado no Instituto Nacional da Propriedade Industrial (INPI) BR512019002197-5, e a avalição de sua usabilidade por enfermeiros especialistas, com abordagem predominantemente quantitativa dos dados. O projeto foi aprovado no comitê de ética C.A.A.E 17039019.1.0000.5546, sob o parecer 4.168.891.

### Desenvolvimento do Sistema

O processo de construção do software seguiu quatro etapas: 1ª) Elaboração de uma revisão sistemática com pesquisas aplicadas cadastradas em bases de dados eletrônicas (PUBMED, SCOPUS, CINAHAL, *Web of Science*) para o levantamento de evidências sobre internação em pronto-socorro (*boarding*); 2ª) Estruturação do arcabouço do sistema; 3ª) Construção dos formulários do sistema; 4ª) Avaliação das informações geradas pelo sistema sob a forma de relatórios, com o cruzamento das informações.

O desenvolvimento do software seguiu o modelo proposto por Sommerville^22^, utilizando uma abordagem sistemática baseada no princípio de quatro atividades fundamentais: Especificação; Desenvolvimento; Validação e Evolução do Software.

A arquitetura utilizada foi o padrão MVC (Model, View and Controller) que permite que o projeto seja trabalhado em camadas, facilitando a manutenção, pois as camadas têm papéis bem definidos. A linguagem de programação Personal Home Page (PHP) foi utilizada para gerar conteúdo dinâmico e para o manuseio do banco de dados, a Structured Query Language (SQL). Foram utilizados os campos de aplicação AD 02 e SD 01 e os programas GI01 – Gerenciador de informação; GI04 – Gerador de relatórios; IA01 – Inteligência artificial; IA02 – Sistemas especialistas.

### Avaliação de Usabilidade

A avaliação da usabilidade do sistema foi realizada utilizando a *System Usability Scale* (SUS), proposta principalmente para a avaliação de aplicativos da web para dois aspectos, a capacidade de aprendizagem e a usabilidade^23^. O questionário foi gerado em língua portuguesa, utilizando o *Free, Multilingual System Usability Scale Questionnaire Generator* que resultou nos seguintes itens:

1. Eu usaria o SIMIPS frequentemente;
2. Achei o SIMIPS desnecessariamente complexo;
3. Eu achei SIMIPS fácil de usar;
4. Eu acho que precisaria de suporte técnico para poder usar o SIMIPS;
5. Eu descobri que as várias funções do SIMIPS estavam muito bem integradas;
6. Eu achei que havia muita inconsistência no SIMIPS;
7. Eu imagino que a maioria das pessoas aprenderia a usar o SIMIPS muito rapidamente;
8. Achei o SIMIPS bastante desconfortável de usar;
9. Eu me senti muito confiante usando o SIMIPS;
10. Eu precisei aprender muitas coisas antes de poder usar o SIMIPS.

O questionário do SUS composto por essas dez questões, misturou itens positivos (questões ímpares) e negativos (questões pares) e, em cada pergunta, o avaliador expressou a magnitude de sua concordância, usando uma escala Likert de 5 pontos com afirmações indo de discordo fortemente (1) para concordo plenamente (5).

Os pesquisadores adicionaram mais uma questão objetiva, que foi excluída do cálculo do escore SUS por não fazer parte do original: Questão 11. O SIMIPS é uma ferramenta que facilita a gestão do PS? E mais uma questão aberta livre: Questão 12. Quais são suas sugestões para melhoria do SIMIPS?

A seleção dos avaliadores levou em consideração a pontuação em pelo menos dois critérios, com enfoque nas áreas assistencial e gerencial de pronto-socorro e/ou segurança do paciente e/ou qualidade hospitalar. Os critérios observados foram: ser enfermeiro, mestre ou doutor com tese ou dissertação nas áreas; ser autor/coautor de artigo científico em periódico revisado por pares; participar/ou ter participado de grupos/projetos de pesquisa; ter experiência de no mínimo um ano, na prática assistencial ou de gestão; ter experiência docente, de no mínimo um ano, na prática assistencial ou gerencial.

O enfermeiro foi considerado profissional-chave na utilização desse sistema, pois está constantemente alimentando os sistemas de informação nos prontos-socorros e gerenciando os fluxos de pacientes.

A seleção dos avaliadores foi realizada por meio de busca ativa na Plataforma Lattes, do Conselho Nacional de Desenvolvimento Científico e Tecnológico (CNPq) (Currículo Lattes e Diretório de Grupos de Pesquisa) e amostragem por bola de neve. Os avaliadores foram contactados via e-mail, por meio de carta convite encaminhada durante o mês de junho de 2020 e, após o aceite formal realizado com assinatura digital do Termo de Consentimento Livre e Esclarecido (TCLE), foram cadastrados no SIMIPS na função Usuário Administrador. Os convites foram enviados semanalmente, até três vezes, com o lembrete para participação e o prazo para o retorno completo da avaliação que se encerrou em 31 de agosto de 2020.

Todos os participantes receberam um tutorial escrito sobre a utilização do sistema e o prontuário de um paciente fictício para cadastrar no sistema e fazer o monitoramento. O objetivo foi simular a alimentação do sistema de forma mais aproximada da realidade possível. Logo após, os avaliadores responderam ao questionário sobre a usabilidade do SIMIPS.

Os avaliadores foram nomeados com a letra J seguido de uma sequência numérica e as questões do questionário SUS nomeadas com a letra Q seguida da sequência numérica.

Os dados quantitativos foram tabulados e analisados no programa Microsoft Excel®. As variáveis numéricas foram expressas em medida de tendência central (média e mediana) e medida de dispersão (desvio padrão). As variáveis categóricas, em frequências absolutas e relativas.

O escore SUS individual foi calculado segundo Brooke^23^ em que, para os itens ímpares, o escore individual é a nota recebida menos 1 e, para os itens pares, o escore é obtido após subtrair 5 da nota recebida. Por fim, multiplica-se a soma de todos os escores por 2,5. O escore SUS total foi calculado obtendo-se a média do escore de cada item e multiplicando-se a soma de todos os escores por 2,5.

Após o cálculo do escore SUS total, o sistema foi classificado da seguinte forma: de 13 a 20,5 (pior imaginável); de 21 a 38,5 (pobre); 39 a 52,5 (mediano); de 53 a 73,5 (bom); de 74 a 85,5 (excelente); e de 86 a 100 (melhor imaginável)^24^.

## RESULTADOS

A revisão da literatura nos apontou que a internação no PS pode provocar redução da qualidade da assistência e omissão de cuidados^6,12^, aumento da mortalidade e aumento do tempo de permanência hospitalar^25, 26^, vulnerabilidade a ocorrência de eventos adversos^5,7,11,12,14^, redução da qualidade e segurança dos cuidados de enfermagem prestados ao paciente^13,27^ e pode contribuir negativamente para as experiências psicossociais dos enfermeiros^27^.

Sendo assim, foram incorporadas no SIMIPS informações relacionadas e complementares aos achados na literatura referentes a:

- Informações de entrada: informações cadastrais do paciente;
- Avaliação inicial: Dados da classificação de risco, da avaliação médica inicial, Anamnese de história de saúde pregressa, classificação do Índice de Charlson (ICC) e do Grau de Dependência dos Cuidados de Enfermagem (GDCE) pelo Sistema de Classificação do Paciente (SCP), proposto por Perroca e Gaidzinski^28^;
- Monitoramento das movimentações do paciente: Dados sobre transferências intra-hospitalar (áreas de internação ou escalonamento de cuidado dentro do pronto-socorro) e saídas (alta, óbito, transferência extra hospitalar e evasão);
- Dados de gestão: Dados sobre a taxa de ocupação do pronto-socorro e setores de internamento, além do tempo de internação no PS (da recepção até a classificação de risco, duração da classificação de risco, dessa classificação até o primeiro atendimento, depois até a decisão pela internação, o tempo de internação dentro do PS, o tempo de internação nas clinicas e o tempo de internação total);
- Avaliação continuada: Dados sobre o GDCE e o *National Early Warning Score* (NEWS) 2^29^;
- Monitoramento de eventos adversos: a Identificação de Evento Adverso (EA) é feita utilizando os rastreadores identificados pelo *Institute for Healthcare Improvement*^30^ e são categorizados pelo *National Coordinating Council for Medication Error Reporting and Prevention* (NCC MERP) *scale*^31^;
- Relatórios de gestão: são informações, apresentadas por meio de tabelas, em que é possível obter o relatório individual por paciente, o relatório geral por espaço de tempo, relatório geral dos tempos de internação e tempo de internação total, obtidos por meio de gráficos com o cruzamento das variáveis EA *versus* Paciente, EA *versus* ICC, EA *versus* SCP, EA *versus* NEWS, EA *versus* Internações no PS, EA *versus* Internações total, EA *versus* Óbitos, EA *versus* Reinternações, Óbito *versus* Internações no PS, Óbitos *versus* reinternações, Taxa de ocupação do PS *versus* EA.

Essa versão do sistema foi concebida para que os dados sejam alimentados de forma secundária por um profissional da assistência e da gestão do pronto-socorro, sendo que o arcabouço do sistema foi construído em subdivisões de seções, subseções e Fichas de informações. A Figura 1 apresenta o arcabouço do SIMIPS.

**Figura 1:** Arcabouço do SIMIPS.

Para que o sistema possa ser alimentado com mais facilidade, o SIMIPS possui uma tecnologia responsiva que adequa o layout ao tamanho do dispositivo utilizado (celular, tablet ou *desktop*). A Figura 2 mostra o layout da tela inicial nos formatos desktop e celular.

**Figura 2:** *Layout* da tela inicial nos formatos desktop e celular.

SIMIPS: Sistema de Informação e Monitoramento das Internações em Pronto-Socorro; Inter. PS: internação no pronto-socorro; SCP: Sistema de Classificação de Paciente.

Com as informações inseridas no sistema é possível realizar um amplo monitoramento do perfil epidemiológico das internações no pronto-socorro, da gestão de tempo dentro da unidade durante a internação, do escalonamento do paciente internado em consequência da deterioração clínica, da incidência e gravidade de eventos adversos além de favorecer um dimensionamento de enfermagem adequado às necessidades de cuidados dos pacientes internos. Todas essas informações podem ser vistas no formato de gráficos com cruzamentos de covariáveis na seção relatórios. A Figura 3 mostra alguns dos relatórios que podem ser gerados pelo SIMIPS.

**Figura 3:** Exemplos de relatórios gerados no SIMIPS.

Para medir a usabilidade do sistema convidamos 43 avaliadores que atenderam aos critérios de elegibilidade, aceitaram participar da pesquisa 17 e todos concluíram a avaliação no prazo estabelecido.

Na avaliação individual, 59% (n = 10) classificou o SIMIPS como melhor imaginável, 35% (n = 6) classificou como ótimo e 6%(n = 1), como bom. Em seguida calculamos o escore final com média de todos os resultados e obtivemos o escore de 86,5 que classifica o software como melhor imaginável.

A caracterização dos avaliadores e a classificação pelo questionário de usabilidade está descrita na Tabela 1. Os valores atribuídos por eles a cada pergunta do questionário estão apresentados na Tabela 2. Adicionamos na tabela a questão 11 (Q11) inserida pelos pesquisadores.

**Tabela 1:** Características dos Avaliadores do Sistema de Informação e monitoramento das Internações em Pronto-Socorro e Classificação pelo questionário de usabilidade (n = 17).

Idade (em anos)	
Média (DP)	34 (7)
Mediana (Min-Máx)	36 (26–53)
Sexo	n (%)
Feminino	13 (76%)
Masculino	4 (24%)
Titulação máxima	n (%)
Mestre	8 (47%)
Doutor	4 (24%)
Especialista	5 (30%)
Perfil profissional	n (%)
Docente	4 (29%)
Assistencial	11 (65%)
Docente/Assistencial	2 (11%)
Pontuação (critérios)	n (%)
2	6 (35%)
3	3 (18%)
4	4 (24%)
5	2 (11%)
6	1 (6%)
7	1 (6%)
Classificação	n (%)
86 a 100 (melhor imaginável)	10 (59%)
74 a 85,5 (excelente)	6 (35%)
53 a 73,5 (bom)	1 (6%)
39 a 52,5 (mediano)	0
21 a 38,5 (pobre)	0
20,5 (pior imaginável)	0
Classificação geral	*Score* sus total
	86,5 (melhor imaginável)

**Tabela 2:** Distribuição individual das respostas ao Questionário de Usabilidade.

JUIZ	Q1	Q2	Q3	Q4	Q5	Q6	Q7	Q8	Q9	Q10	Q11
J1	5	1	4	2	4	1	5	1	4	1	5
J2	5	1	5	2	5	2	5	1	5	1	5
J3	5	1	5	1	5	2	4	1	5	1	5
J4	5	2	5	3	4	2	5	2	4	1	5
J5	5	1	4	2	4	1	5	1	4	1	5
J6	5	1	5	4	4	1	5	1	5	1	5
J7	5	2	4	1	5	1	5	1	5	1	5
J8	4	1	4	2	4	2	4	5	5	1	4
J9	5	1	5	2	5	1	5	1	5	2	5
J10	5	2	4	1	4	1	5	2	4	2	5
J11	5	1	5	1	4	1	3	2	4	1	5
J12	4	1	2	1	4	2	4	2	4	1	5
J13	5	2	4	4	5	1	5	2	4	3	5
J14	4	3	3	2	5	2	2	1	4	3	4
J15	4	2	4	3	5	1	3	3	5	1	5
J16	5	1	5	2	5	1	5	1	5	1	5
J17	5	1	5	2	5	2	5	1	5	1	5

Na Q11, em que questionamos se o SIMIPS é uma ferramenta que facilita a gestão do PS, obtivemos como resposta, concordo totalmente (n = 15) e concordo parcialmente (n = 2).

Em resposta à última pergunta, obtivemos sugestões de melhorias para o SIMIPS relacionadas a: 1) tornar o layout mais atrativo; 2) quando possível, integrá-lo sistema de informação hospitalar, para facilitar o preenchimento de informações como sinais vitais, dados socioeconômicos, as movimentações e o tempo de permanência; 3) inserir um filtro de preenchimento automático para o CID 10; 4) Inserir a opção de visualização do formulário preenchido, antes de salvar, e inserir a opção “editar” e 5) Colocar na página inicial a lista com os nomes dos pacientes internados no momento, ao invés do gráfico de taxa de ocupação x eventos adversos.

## DISCUSSÃO

Os Sistemas de Informação em Saúde (SIS) têm como finalidade auxiliar a melhoria na qualidade de atendimentos dos pacientes e profissionais da saúde, bem como a gestão da saúde por meio da análise dos custos, benefícios e da redução de erros médicos^32^.

Nesse sentido, o SIMIPS foi concebido como um sistema de informação local (institucional), mas com possibilidade de integrar-se a outros sistemas de informação locais, gerando bases regionais, além de gerar relatórios que podem ser inseridos em bancos de informações locais, regionais e nacionais.

Um pressuposto implícito no desenvolvimento de um Sistema de Informação Hospitalar é a habilidade de fornecer o dado completo, exato e no momento adequado, para que o profissional possa desempenhar sua tarefa com maior qualidade e com melhor razão custo/benefício^17^.

O SIMIPS teve a sua usabilidade avaliada por enfermeiros com predomínio de mestres (n = 8), atuantes na assistência em pronto-socorro (n = 11) e que atenderam a mais de 2 critérios de inclusão (n = 11), demonstrando capacidade científica e técnica dos avaliadores recrutados.

O questionário SUS avaliou a usabilidade do SIMIPS por meio da percepção do usuário, sendo classificado como melhor imaginável (escore 85,6) na avaliação total e não obtendo nenhuma avaliação negativa (mediano, pobre e pior imaginável).

Analisando as respostas dos usuários nos dois fatores elencados pelo SUS, usabilidade (questões 1, 2, 3, 5, 6, 7, 8 e 9) e aprendizagem (questões 4 e 10), observamos nas questões negativas 2, 6 e 8, um predomínio das respostas discordo fortemente e discordo parcialmente (Q2 n = 16, Q6 n = 17 e Q8 n = 15), onde apenas um usuário concordou fortemente que o SIMIPS seria bastante desconfortável de usar. Nas questões positivas de usabilidade (1, 3, 5, 7 e 9) houve um predomínio das respostas concordo plenamente e concordo parcialmente (Q1 n = 17, Q3 n = 15, Q5 n = 17, Q7 n = 14, Q9 n = 17).

Nas questões que avaliaram a facilidade de aprendizagem sobre o SIMIPS, as respostas concordo plenamente e concordo parcialmente também predominaram (Q4 n = 13 e Q10 n = 15), o que denota que o SIMIPS pode ser implementado em um pronto-socorro com alta possibilidade de aceitação.

A implementação de um Sistema Eletrônico de Saúde é desafiadora, pois causa mudanças e pode despertar resistência, portanto, o fluxo de informações deve ser desenvolvido para facilitar seu uso, a compreensão e a comunicação da equipe^33^.

O predomínio das respostas concordantes para as questões positivas e discordantes para as negativas, tanto no quesito usabilidade quanto em aprendizagem, apontam a possibilidade de fácil aceitação do SIMIPS como uma ferramenta de gestão, visto que também foi apontado por todos os avaliadores que o software facilita a gestão do PS.

Considerando que o SIMIPS é uma ferramenta nova, apresenta algumas limitações, como por exemplo, o preenchimento secundário, que precisa da adesão da equipe de saúde e dos gestores do pronto-socorro, um setor que, especificamente, tem como característica o fluxo intenso de informações e pacientes. Contudo, em instituições onde já se utiliza um sistema de informação, como o prontuário eletrônico, é possível integrar essas informações ao SIMIPS, agilizando seu preenchimento. Outra limitação do sistema é seu uso em urgências pediátricas, visto que foi desenvolvido para utilização em pronto-socorro adulto.

Porém, não foi identificada na literatura menção a outro SIS ou de monitoramento que objetive avaliar e gerenciar as internações no PS, destacando o caráter inovador do SIMIPS. Desse modo, a utilização do SIMIPS como ferramenta de monitoramento pode subsidiar pesquisas futuras sobre o impacto da permanência prolongada no pronto-socorro, na gestão de recursos humanos e materiais, na gestão de leitos e na qualidade de vida e no trabalho dos profissionais de enfermagem.

Como ferramenta de gestão, o monitoramento gerado pelo SIMIPS apresentará indicadores de qualidade da assistência no pronto-socorro aos pacientes internados, além de fornecer avaliação dos usuários sobre os serviços prestados durante a internação, informações que podem nortear as mudanças nos processos de trabalho voltados para segurança do paciente.

## CONCLUSÃO

O SIMIPS foi considerado pelos avaliadores uma ferramenta de fácil usabilidade e importante utilidade na gestão do pronto-socorro. No entanto, alguns ajustes foram apontados para melhorar a capacidade de recepção de informações que melhorem a agilidade no preenchimento e no gerenciamento de informações. É importante salientar que o SIMIPS foi desenvolvido para uso em PS adulto e que serão necessárias adaptações para sua utilização em pediatria, bem como para atender especificidades regionais.

Contudo, o seu projeto inicial mostrou-se capaz de gerar dados de gestão e indicadores assistenciais que podem nortear ações de enfrentamento da superlotação e das internações em pronto-socorro, propondo estratégias de mitigação dos efeitos da superlotação na qualidade do cuidado.
